# Microglial cell dysregulation in brain aging and neurodegeneration

**DOI:** 10.3389/fnagi.2015.00124

**Published:** 2015-07-20

**Authors:** Rommy von Bernhardi, Laura Eugenín-von Bernhardi, Jaime Eugenín

**Affiliations:** ^1^Department of Neurology, Faculty of Medicine, Pontificia Universidad Católica de ChileSantiago, Chile; ^2^Laboratory of Neural Systems, Department of Biology, Faculty of Chemistry and Biology, Universidad de Santiago de Chile (USACH)Santiago, Chile

**Keywords:** Alzheimer’s disease, glia, mitochondria, neurodegenerative diseases, neuroinflammation, oxidative stress, reactive oxygen species, transforming growth factor-β

## Abstract

Aging is the main risk factor for neurodegenerative diseases. In aging, microglia undergoes phenotypic changes compatible with their activation. Glial activation can lead to neuroinflammation, which is increasingly accepted as part of the pathogenesis of neurodegenerative diseases, including Alzheimer’s disease (AD). We hypothesize that in aging, aberrant microglia activation leads to a deleterious environment and neurodegeneration. In aged mice, microglia exhibit an increased expression of cytokines and an exacerbated inflammatory response to pathological changes. Whereas LPS increases nitric oxide (NO) secretion in microglia from young mice, induction of reactive oxygen species (ROS) predominates in older mice. Furthermore, there is accumulation of DNA oxidative damage in mitochondria of microglia during aging, and also an increased intracellular ROS production. Increased ROS activates the redox-sensitive nuclear factor *kappa* B, which promotes more neuroinflammation, and can be translated in functional deficits, such as cognitive impairment. Mitochondria-derived ROS and cathepsin B, are also necessary for the microglial cell production of interleukin-1β, a key inflammatory cytokine. Interestingly, whereas the regulatory cytokine TGFβ1 is also increased in the aged brain, neuroinflammation persists. Assessing this apparent contradiction, we have reported that TGFβ1 induction and activation of Smad3 signaling after inflammatory stimulation are reduced in adult mice. Other protective functions, such as phagocytosis, although observed in aged animals, become not inducible by inflammatory stimuli and TGFβ1. Here, we discuss data suggesting that mitochondrial and endolysosomal dysfunction could at least partially mediate age-associated microglial cell changes, and, together with the impairment of the TGFβ1-Smad3 pathway, could result in the reduction of protective activation and the facilitation of cytotoxic activation of microglia, resulting in the promotion of neurodegenerative diseases.

## Introduction

Aging is a complex process of cumulative changes. A key hallmark is the progressive decline in physiological functions and behavioral capacity, which is observed at various levels of the organism, in particular at the central nervous system (CNS; Smith et al., 2005). These changes can lead to altered behavior, memory impairment, or loss of several control functions (Lipsitz and Goldberger, 1992; Lipsitz, 2002; Glenn et al., 2004). In addition, some responses of the immune system, in special related to adaptive immune system, also decline with age, increasing the susceptibility to infections and cancer. By contrast, other immune responses are exacerbated, facilitating the onset of autoimmune diseases (Yung and Julius, 2008) or the generation of a mild chronic neuroinflammation mediated by the dysregulation of the innate immune system, as will be discussed here. Therefore, aging can affect several tissues and processes, leading to highly complex functional changes.

Microglia undergoes several age-related changes that contribute to the generation of a chronic mild inflammatory environment, including an increased production of inflammatory cytokines and the production of reactive oxygen species (ROS). These changes have been linked to the appearance of cognitive deficits and the onset of chronic neurodegenerative diseases. Therefore, it has been proposed that aging of microglia could contribute to other age-associated brain changes and cognitive decline (Conde and Streit, 2006a, b; Streit, 2006; von Bernhardi, 2010; Aguzzi et al., 2013; Kettenmann et al., 2013).

## Normal Brain Aging

Several structural and functional changes associated with normal brain aging have been reported. Brain mass decreases in the order of 2 to 3% per decade after the age of 50. Individuals that are 80 years or older, brain mass is reduced by 10% compared with that of young adults (Drachman, 2006). Magnetic resonance imaging (MRI) and voxel-based morphometry (VBM) show that age specially affects the volume of gray and white matter at prefrontal, parietal, and temporal areas (Ge et al., 2002; Sowell et al., 2003; Salat et al., 2004). Complex learning abilities, such as dual tasks (ea. memorizing a word list while walking), show a progressive decrease during aging (Lindenberger et al., 2000; Salat et al., 2005). Nevertheless, cognitive decline in aging is highly variable; many older people keep intact their cognitive abilities (Shock et al., 1984) until advanced ages.

**Figure 1:**
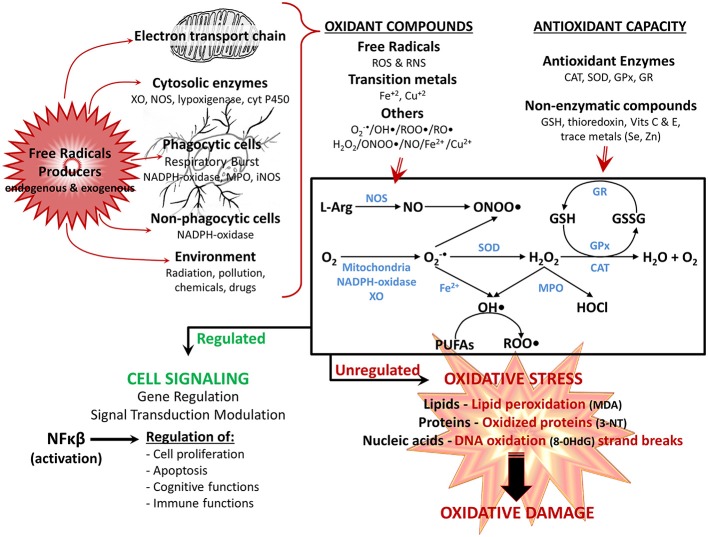
**Reactivespecies participate in normal cellular function orin pathological mechanisms depending on their overproduction.** Reactive oxygen species (**ROS**) and reactive nitrogen species (**RNS**), are produced through several mechanisms by the cell: the electron transport chain in mitochondria, various cytosolic and membrane enzymes (i.e., xanthine oxidase (**XO**), nitric oxide synthase (**NOS**), NADPH oxidase complex, etc.), as well as exogenously provided by the environment. At the same time, cells have several antioxidant defense mechanisms for detoxifying ROS and RNS, including enzymes (i.e., superoxide dismutase (**SOD**), catalase (**CAT**), glutathione peroxidase (**GPx**), glutathione reductase (**GR**) and nonenzymatic antioxidants (i.e., reduced glutathione (**GSH**), vitamins **E** and **C**. The main generation pathways of ROS and RNS are also shown: the reduction of O_2_ occurs by diverse mechanisms (i.e., mitochondria, XO, NADPH-oxidase complex) leading to formation of superoxide anion (**O_2_^•-^**); which is easily transformed to hydrogen peroxide (**H_2_O_2_**) either nonenzymatically or by SOD. H_2_O_2_ is converted to H_2_O by CAT, or by GPx, which together with the GR regenerate GSH. In addition, under stress conditions and high concentration of transition metal (i.e., iron ions—**Fe**), O_2_ •- can generate hydroxyl radical **(OH•)**, which in turn can react with polyunsaturated fatty acids (**PUFAs**) and generate peroxyl radical (**ROO•**). Finally, O_2_ •- can react with nitric oxide (**NO**; depending on NOS), producing the highly reactive peroxinitrite (**ONOO•**) anion, whereas H_2_O_2_ is converted to hypochlorous acid (**HOCl**) by myeloperoxidase (**MPO**). The balance between oxidants compounds and antioxidant defense determines the end result. Optimal physiologic levels leads to beneficial effects, with ROS and RNS acting as second messengers in intracellular signaling cascades (modulation of gene regulation and signal transduction pathways, mainly by activation of NFκB), regulating several physiological functions (i.e., cognitive and immune functions). However, when overproduction of ROS/RNS is higher than the antioxidant system, the equilibrium status favors oxidant vs. antioxidant reactions, leading to **oxidative stress**, in which ROS/RNS have harmful effects, because of their reaction with various macromolecules (lipids, proteins and nucleic acids), contributing to cellular and tissue oxidative damage, and the development of age-related impairments. **Oxidation products**: 3-NT, 3-nitrotyrosine; 8-OHdG, 8-hydroxy-2-deoxyguanosine; malondialdehyde (**MDA**); alkoxyl radical (**RO•**).

At the cellular level, shortening of telomeres and activation of tumor suppressor genes, as well as accumulation of DNA damage, oxidative stress, and mild chronic inflammatory activity are characteristic of aging cells. Various tissues, including the brain show an imbalance between pro- and anti-inflammatory cytokine levels. In addition, potentially damaging mediators, such as cytokines, radical species (Figure 1), and eicosanoids among others, are produced in response to the exposure to physical, chemical or biological agents, such as ionic radiation, pollutants, pathogens, etc. (Dröge and Schipper, 2007; Vijg and Campisi, 2008). Both humans and mice show decreased levels of interleukin 10 (IL10; Ye and Johnson, 2001), and increased levels of tumor necrosis factor α (TNFα) and IL1β in the CNS (Lukiw, 2004; Streit et al., 2004a), and IL6 in plasma (Ye and Johnson, 2001; Godbout and Johnson, 2004). In addition, increased transforming growth factor β1 (TGFβ1) mRNA a key cytokine regulator, has been observed in the brain of aged mice and rats (Bye et al., 2001).

At the same time, several changes induced by an aged micro-environment, such as increased systemic inflammation, increased permeability of the blood-brain barrier (BBB), and degeneration of neurons and other brain cells, could contribute to the production of ROS. It has been proposed that BBB permeability increases in aged animals (Blau et al., 2012; Enciu et al., 2013), facilitating perhaps infiltration by monocytes releasing mitochondria-generated ROS. An age-related increase in the number of CD11b^+^ CD45^high^ cells, compatible with infiltrated monocytes, has been reported in the brain of aged rats (Blau et al., 2012). Likewise, expression levels of chemotactic molecules, such as interferon-inducible protein 10 (IIP10) and monocyte chemotactic protein-1 (MCP-1), are increased in the hippocampal region (Blau et al., 2012).

## Glial Cells, Neuroinflammation and Oxidative Stress

Neuroinflammation is choreographed by microglia and astrocytes, and is defined by increased levels of a complex arrangement of mediators, including IL1β, TNF*α* and TGFβ, all of which are increased in aged individuals (McGeer and McGeer, 2001; von Bernhardi, 2007; von Bernhardi et al., 2010). Microglia are the brain resident macrophages (Hemmer et al., 2002; Ransohoff and Perry, 2009; Rivest, 2009) providing its first line of defense. In the brain of healthy adults, microglia are slender ramified cells that constantly survey brain parenchyma (Davalos et al., 2005; Nimmerjahn et al., 2005). When stimulated, microglia activate, enlarge their cell body (Nimmerjahn et al., 2005; Frank-Cannon et al., 2009) and change their functional properties (Liu et al., 2001; von Bernhardi and Eugenín, 2004; Lue et al., 2010). Microglia sense and act on a broad range of stimuli, including autoimmune injury, infection, ischemia, toxic insults and trauma (Streit, 2002; Kim and de Vellis, 2005; Schwab and McGeer, 2008; Lue et al., 2010; von Bernhardi et al., 2010). They recognize a broad spectrum of molecular targets, such as glycolipids, lipoproteins, nucleotides, peptides, (Nakamura, 2002; van Rossum and Hanisch, 2004; Pocock and Kettenmann, 2007), abnormally processed, modified or aggregated proteins (e.g., Aβ), inflammatory cytokines, and damaged neurons, which are the strongest inducers of microglia activation (Nakamura, 2002; Hanisch and Kettenmann, 2007; Ransohoff and Perry, 2009; Lue et al., 2010; Schuitemaker et al., 2012). Depending on the stimuli, microglia undergoes different activation patterns (Gordon, 2003; Martinez et al., 2008; Mosser and Edwards, 2008). They include (i) classical M1 activation, which can associate with cytotoxicity, (ii) alternative phagocytic/neuroprotective M2 activation (Gordon, 2003; Martinez et al., 2008) or (iii) regulatory activation (Mosser and Edwards, 2008). Thus, activated microglia show a continuum spectrum of activation patterns, resulting in the expression of different cytokines and cytokine receptors (Town et al., 2005).

Commitment to the M1 macrophage lineage (Satoh et al., 2010) is defined by the activation of a member of the interferon-regulatory factor (IRF) family. IRF5 activates genes encoding for inflammatory cytokines, such as TNFs, IL6, IL12 and IL23, and tumor suppressors (Ouyang et al., 2007; Krausgruber et al., 2011). M2 polarization is controlled by IRF4 (Satoh et al., 2010; Krausgruber et al., 2011). Cyclic AMP-response element binding protein (CREB)–mediated induction of transcription factor C/EBPβ upregulates M2-specific genes (Ruffell et al., 2009), whereas activation of transcription factor nuclear factor *kappa*-light-chain-enhancer of activated B cells (NFκB)-p50 is associated with the inhibition of M1-activation genes (Porta et al., 2009). Secretion of IL4, IL10 and TGFβ by M2-activated macrophages, promote humoral immune responses and down-regulate M1-mediated responses, inhibiting several inflammatory functions (Town et al., 2005). Originally, it was thought that M2 activation resulted in protective functions. However, there is evidence that M2 cytokines such as IL4, IL5, IL9, and IL13 also result in the induction of some chronic inflammatory processes (Wynn, 2003). As for regulatory macrophages; they appear to arise at later stages of adaptive immune responses, being their primary role limiting inflammatory activation (Mosser, 2003). Regulatory macrophages appear to be generated through several signaling pathways, involving extracellular signal-regulated kinases/mitogen-activated protein kinases (ERK/MAPK; Lucas et al., 2005; Mosser and Edwards, 2008).

Microglia are activated in nearly all CNS diseases (Kreutzberg, 1996; Hanisch and Kettenmann, 2007; Neumann et al., 2009), producing and secreting a broad spectrum of inflammatory mediators, such as eicosanoids, cytokines (Nakamura, 2002; Kim and de Vellis, 2005; Tichauer et al., 2007), chemokines, ROS, nitric oxide (NO·), small metabolites, proteases (ea. α-antichymotrypsin and α-antitrypsin), and inflammatory markers (ea. serum amyloid P and C-reactive protein; Li et al., 2007; Tichauer et al., 2007; Neumann et al., 2009; Lue et al., 2010). Those inflammatory mediators regulate innate immune defense and have profound effects on neuronal properties, modifying synaptic function (Selkoe, 2002; Di Filippo et al., 2008). In addition, microglia can also induce bystander damage of neurons, especially under conditions of strong or long lasting stimulation, and depending on the environmental context (Li et al., 2007; von Bernhardi, 2007). In fact, cytotoxic activation of microglia is associated with neuronal loss and decline of cognitive and neurobehavioral function (Cagnin et al., 2001; Kim and de Vellis, 2005; Block et al., 2007). Nevertheless, microglia also secretes trophic factors and modulator cytokines, being active partners in neuroprotection.

Neuroinflammation establishes a complex interaction with oxidizing agents through redox sensors present in enzymes, receptors, and transcription factors. Those factors affect neuron-glia crosstalk and neuronal function (Liu et al., 2012), resulting later in neurodegenerative changes (Raj et al., 2014). Signal transduction of various cytokines, themselves critical mediators of oxidative stress, neuroinflammation, and even neurodegenerative changes, are modified by the redox status (Mrak and Griffin, 2005; Kierdorf et al., 2010). Oxidative stress, a result of the equilibrium between production and detoxification of radical species (Figure 1), further increases inflammatory cytokines, creating a vicious cycle (Rosales-Corral et al., 2010), and affects the maintenance of cellular homeostasis and cell survival (Satoh and Lipton, 2007).

**Figure 2:**
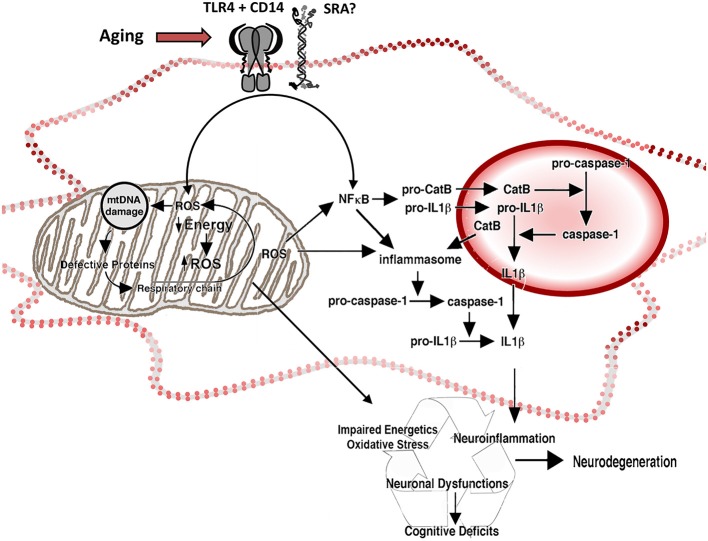
**Reactive oxygen species and inflammation in the aged microglia.** Representation of the participation of mitochondria and lysosomes in the increased production of ROS and inflammatory cytokines by aged microglia. Increased intracellular ROS activate redox-sensitive NFκB through a pathway mediated by mitochondrial ROS (associated with decreased energetic production and increased release of ROS by the electron transport chain) and a ROS-independent pathway, potentiating neuroinflammation. The activation of NFκB induces production of pro-CatB and pro-IL1β, and the activation of inflammasome in the cytoplasm. Pro-CatB is processed into CatB in the lysosome, which in turn, mediates the activation of pro-caspase-1 to caspase 1 and increases the processing of pro-IL1β, releasing increased amounts of IL1β both in the phagolysosome and the cytoplasm, as well as potentially potentiate apoptosis. Changes on the expression of pattern recognition receptors, like TLR4 CD14 and SRA, result in changes on neuroinflammatory activation and oxidative stress by activating NFκB and the release of ROS.

Mitochondria were often thought to be the main responsible for ROS overproduction and oxidative stress. However, NADPH oxidase (NOX) enzymes participation is also an important ROS-generating system (Bordt and Polster, 2014). Activation of the phagocyte NADPH oxidase (NOX2) in microglia, plays a role in neuroinflammation, but appears also to contribute to neuronal death under pathologic conditions (Qin et al., 2013; Jiang et al., 2015). Moreover, ROS production can also depend on other NOX isoforms, which are detected also in astrocytes and neurons (Nayernia et al., 2014). Whereas ROS derived from normal NADPH oxidase function is required for processes such as neuronal signaling, memory, and central homeostasis (Jiang et al., 2015), overproduction of ROS contributes to excessive oxidative stress, resulting in neuronal dysfunction and neurotoxicity (Zhang et al., 2014). ROS regulates several signal transduction pathways, including for some trophic factors and hormones. NF*κ*B is a transcription factor activated by ROS and inflammatory mediators that participates both in protective and deleterious responses, depending on the context of stimulation that will result in the co-activation of various signaling pathways. It activates genes regulating cellular survival, growth, differentiation, inflammation, and cell death. Under non-stimulated conditions, NFκB is kept inactive by IκB (inhibitor of κB) in the cytoplasmic compartment. High concentrations of ROS inactivate NFκB through oxidation of its p50 subunit, inhibiting its DNA binding. In contrast to the inhibitory effect of high ROS levels, moderate levels of ROS lead to the sequential phosphorylation, polyubiquitination and degradation of IκB, allowing the activation of NFκB (Figures 1, 2). Once activated, and depending on the context, NFκB plays a pro-survival role by inhibiting c-Jun N-terminal kinases/stress-activated protein kinase (JNK) and caspase cell death pathways and upregulating transcriptional activation of anti-apoptotic proteins and genes involved in decreasing mitochondrial ROS (mtROS), especially those coding for manganese superoxide dismutase (MnSOD; Patten et al., 2010). TNF*α* also activates NFκB associated with neuroprotection against β-amyloid (Aβ) neurotoxicity *in vitro* (Barger et al., 1995), and NFκB activates anti-apoptotic responses and protects neurons from excitotoxicity and ischemic brain injury (Pennypacker et al., 2001; Bhakar et al., 2002; Mattson, 2005).

On the other hand, NFκB activation can also be detrimental. NFκB has a key role in the initiation and amplification of inflammation through its response to inflammatory stimuli mediated by TNF*α* or IL1, leading to the induction of several cytokines and chemokines. Activation of NFκB and MAPK pathways are conspicuous in oxidative stress- (Chen et al., 2009; Chongthammakun et al., 2009) and Aβ-induced (Song et al., 2004) neuronal cell death. In addition to NFκB, other transcription factors are activated by inflammatory conditions, such as peroxisome proliferator-activated receptor gamma (PPARγ) and signal transducer and activator of transcription (STAT-1) and have also been implicated in Alzheimer’s disease (AD; Sastre et al., 2006; Cho et al., 2007).

The brain is particularly vulnerable to oxidative stress. Vulnerability depends on its: (i) high oxygen metabolic rate (consumes approximately 20% of the total consumption of oxygen of a mammal), (ii) high dependence on oxidative metabolism for obtaining energy, (iii) high content of iron, an endogenous catalyzer for the generation of ROS and reactive nitrogen species (RNS), (iv) lower content of antioxidant enzymes compared with other organs (Floyd and Hensley, 2002; Mattson et al., 2002); and (v) low ability to eliminate mutations not removed by cell replacement as consequence of the post-mitotic nature of neurons. Aged, or injured brains of any sort, show oxidative modifications in nucleic acids, proteins, lipids, and sugars (Figure 1). Several of those oxidative damage and changes result in a loss of function (Lovell et al., 2001; Halliwell, 2006).

## Age-Related Changes of Microglia

Microglial cell changes have been documented in aging. However, many of those changes are also observed in neurodegenerative conditions. Thus, it is still unclear whether these changes are reactive to the underlying pathophysiology. Although there is an agreement on the fact that degenerative diseases are not the natural continuous progression of age-related decline, both aging and neurodegenerative disease appear to be highly multifactorial conditions that also share many relevant factors. Aging is a mayor risk factor for the development of many neurodegenerative diseases. Furthermore, neuroinflammation and oxidative stress (both reportedly associated with non-pathological aging in humans and animal models) are common features for several disease phenotypes. Studies in cell cultures and animal models suggest the existence of altered activation states and cellular senescence in the aged brain. Not only aging appears to be a key risk factor for neurodegenerative as well as other chronic diseases (Mosher and Wyss-Coray, 2014; Cho et al., 2015), but the presence of those diseases potentiate also the appearance of aging and senescence related markers (Baron et al., 2014; Mosher and Wyss-Coray, 2014; Bachstetter et al., 2015).

There is high heterogeneity of microglia in various neurodegenerative diseases and those phenotypes share common characteristics with aging (Bachstetter et al., 2015) as well as the pattern of microglia gene expression is shared by aging and neurodegenerative conditions (Holtman et al., 2015). Moreover, many of the changes described in aged microglia represent changes that occur during aging; meaning that, they do not appear when reaching a certain age threshold, but they change through life, as the individual ages. Analysis of transcriptome data from postmortem studies of frontal cortex from 381 healthy individuals with ages spanning from young teenagers to people older than 80 years of age, show that microglia gene markers assemble into a transcriptional module in a gene co-expression network (Wehrspaun et al., 2015), whose expression pattern show a negative correlation with age. Genes that encode microglia surface receptors for neuron and/or microglia crosstalk are especially affected. In addition, they found that microglia are controlled by brain-expressed transcription factors, including *RUNX1*, *IRF8*, *PU.1*, and *TAL1* (Kierdorf and Prinz, 2013), which are master regulators for the age-dependent microglia module. As the authors highlighted, identification of age-dependent gene modules in adulthood are relevant for understanding critical periods for susceptibility to late-onset diseases (Wehrspaun et al., 2015).

**Figure 3:**
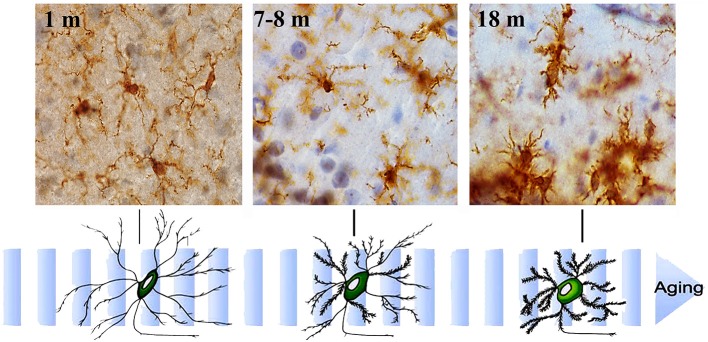
**Aging-related morphological changes of microglia.** Microglial cell morphology changes with aging. Immunohistochemistry for Iba-1 (a constitutive identity marker for monocyte-macrophage cells) and counterstaining with hematoxylin of hippocampal sections from animals of different ages (1- to 18-month old). Microglia obtained from young mice have a small cell body and very long and slender ramifications. As mice age, microglia gradually show bigger cell bodies and progressively shorter and thicker cell processes.

Senescent microglia display morphological changes (Figure 3), with fewer and shorter processes, increased soma volume, and formation of spheroid swellings, which is referred as “dystrophic microglia” (Streit et al., 2004b; Conde and Streit, 2006a, b; Streit, 2006; Flanary et al., 2007). Microglia co-localize with neurodegenerating neurons, and show clumping, with loss of their homogeneous tissue distribution, and accumulation of phagocytic inclusions (Hart et al., 2012; Tremblay et al., 2012; Hefendehl et al., 2014). Live imaging shows that the dynamic response of microglia to injury changes with age. Young microglia increase their motility and extend ramifications rapidly when exposed to ATP, an injury-associated signal, or to a focal tissue injury. In contrast, aged microglia are less dynamic and ramified and further reduce their dynamism when exposed to ATP. On the other hand, disaggregation of aged microglia from the site of injury becomes slow, indicating that aged microglia tend to show sustained responses (Damani et al., 2011). Both in aging (Flanary and Streit, 2004) and in AD (Flanary et al., 2007), microglia show telomere shortening and decreased telomerase activity, which are speculated to be one of the factors underlying the diminution of some functional activities, such as clearance (phagocytosis plus effective removal of the compounds) and basal proliferation (Harry, 2013). Reduced microglia replication could also result in a depletion of healthy microglia, favoring the participation of more senescent and dysfunctional cells (Mosher and Wyss-Coray, 2014).

**Figure 4:**
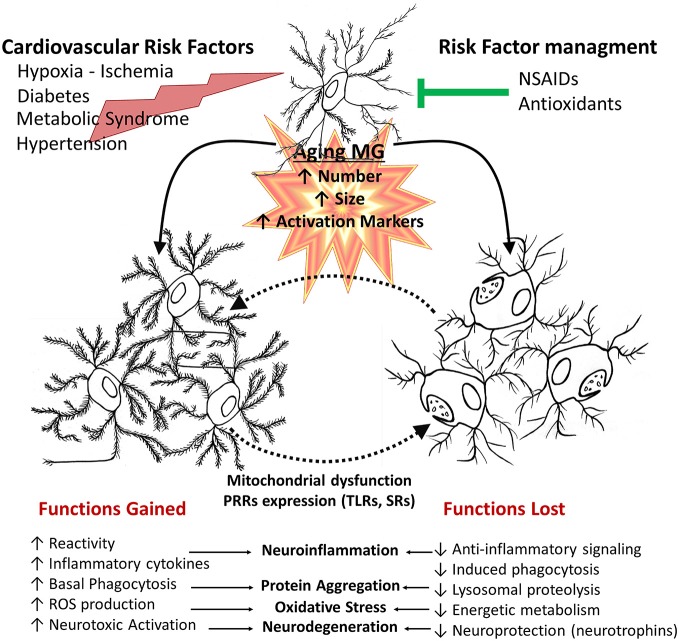
**Age-related changes of microglial cell function.** In aged brains, there is an increased number, size and activation of microglia. This is affected by additional systemic pathophysiological changes associated with other age related changes, environmental factors and disease processes, such as cardiovascular risk factors and metabolic syndrome or injuries. Deleterious processes further promote an inflammatory environment, increasing cytotoxic microglial cell activation, whereas risk factor management and pharmacological interventions can promote a healthy aging. Aged microglia changes depend both on gained and lost functions. They have increased basal phagocytic activity, although a reduced capacity to induce phagocytosis when stimulated, together with reduced lysosomal activity, resulting in a decreased clearance activity. Microglia also shows an increased production of inflammatory cytokines and reactive species. Those changes result in a shift of balance towards decreased protective functions and an increased neurotoxicity.

Activated microglia are the primary cellular source of both inflammatory molecules and oxidative products (Figure 4). (Pawate et al., 2004; Qin et al., 2005b; Hayashi et al., 2008). Microglia from aged brains show increased basal production of IL6 and enhanced lypopolysaccharide (LPS)-induced IL6 and IL1β, compared with microglia from young mice brains in culture (Ye and Johnson, 1999; Sierra et al., 2007). They appear to be activated also under normal physiological conditions. In aging, mild stimulatory events or minor injuries, otherwise easily solved, could induce damage and initiate a disease process. TGFβ1 is a strong regulator of neuroinflammation and cytotoxicity and its signaling pathway could be part of the switch mechanism from protective to deleterious activation of microglia. Its downstream canonical signaling involves the Smad pathway, which transduce extracellular signals from ligands acting as transcription factors (Derynck and Zhang, 2003), as well as a complex Smad independent signaling (Weiss and Attisano, 2013). TGFβ1 secreted by hippocampal neurons and astrocytes regulates microglial cell activation, attenuating the release of inflammatory cytokines and reactive species (Chen et al., 2002; Mittaud et al., 2002; Herrera-Molina and von Bernhardi, 2005; Herrera-Molina et al., 2012), protecting neuronal cells *in vitro* (Hu et al., 1995; Lieb et al., 2003; Herrera-Molina and von Bernhardi, 2005) and promoting microglia-mediated Aβ phagocytosis and degradation (Wyss-Coray et al., 2001). These regulatory effects of TGFβ1 are mediated by Smad3-dependent mechanisms (Flores and von Bernhardi, 2012; Tichauer and von Bernhardi, 2012), as well as the reported inhibition of lipopolysaccharide (LPS)-induced macrophage and microglial activation (Werner et al., 2000; Le et al., 2004). TGFβ1 Smad3 pathway also participates in the inhibition of the production of radical species induced by inflammatory stimuli and in the induction of amyloid-β (Aβ) phagocytosis *in vitro* (Tichauer and von Bernhardi, 2012).

TGFβ1 levels are elevated in aged individual (Blobe et al., 2000; Tichauer et al., 2014). However, recent reports show that induction of the Smad3 pathway by inflammatory conditions is decreased in normal aging (Tichauer et al., 2014). Interestingly, this signaling pathway is impaired in AD patients and mouse models for AD, resulting in Aβ accumulation, Aβ-induced neurodegeneration, and neurofibrillary tangle formation (Tesseur et al., 2006; Ueberham et al., 2006). Evidence gathered over the last two decades indicate that TGFβ signaling impairment often lead to neuroinflammation, neuronal dysfunction and neurodegenerative changes, and could be involved in the pathogenesis of neurodegenerative diseases (Tesseur and Wyss-Coray, 2006). Given the complex signaling pathway activated by TGFβ, which in addition to the Smad pathway also activates Smad-independent signaling, including ERK/MAPK, P38 MAPK, JNK, and PI3K (Derynck and Zhang, 2003; Weiss and Attisano, 2013), a decreased activation of Smad3 in an environment presenting elevated levels of TGFβ, as observed in aging, could result in an increased activation of MAPKs and PI3K, which are signaling pathways also involved in inflammatory activation. Such an imbalance on the signaling activated by TGFβ could explain, at least partially, the maintenance of increased levels of microglial cell activation, oxidative stress and mild neuroinflammation, although TGFβ1, one of the main regulatory cytokines decreasing inflammatory activation, is increased in aged mice (Tichauer et al., 2014). Those results indicate that TGFβ1-Smad3 signaling could be a therapeutic target for AD treatment.

**Figure 5:**
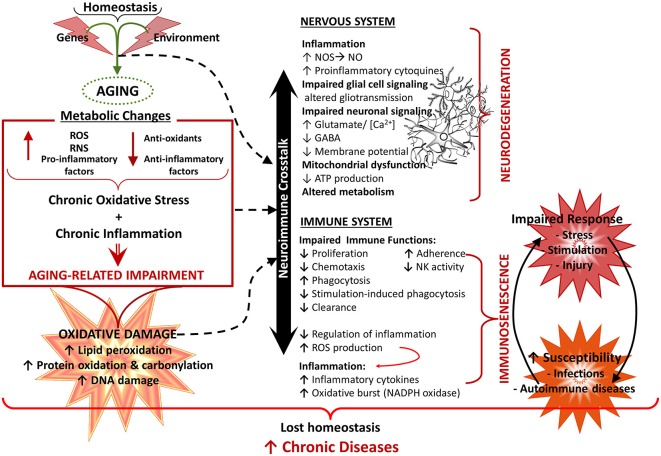
**Aging of the nervous and immune system and the neuroimmune crosstalk.** Healthy aging of the nervous and immune systems depend both on genetic and environmental (lifestyle) factors. Aging is associated with a state of low grade chronic oxidative stress and inflammation (with production of reactive mediators and inflammatory compounds and a decreased antioxidant and anti-inflammatory capacity), which appear to be the cause of an important part of age-related deterioration of the nervous and the immune systems, as well as of the neuroimmune communication. Because of their complex functions, the central nervous system (CNS) and the immune system are especially vulnerable to oxidative damage (i.e., lipid peroxidation, protein oxidation, DNA damage), which contributes to oxidative stress and inflammation. Age-related changes in the immune function, known as immunosenescence, results in increased susceptibility to infections and cancer, inflammation and autoimmune diseases. In the CNS, oxidative stress has a negative impact on function, leading to mitochondrial dysfunction and impaired energetic metabolism, altered neuronal and glial signaling. There may be disruption of the cycle glutamate-glutamine and increased levels of neuronal calcium, which are involved in mechanisms of neuronal damage leading to loss of function, excitotoxicity and apoptosis. In addition, dysfunction of the neuron-glia crosstalk leads to a chronic neuroinflammation, which promotes a prolonged activation of microglia and further induction of dysfunction and degenerative changes. All these alterations contribute to functional decline and the development of neurodegenerative diseases.** NO**, nitric oxide; **NOS**, nitric oxide synthase; **RNS**, reactive nitrogen species; **ROS**, reactive oxygen species.

Another alternative is that stimuli that normally would trigger a protective response, in conditions of age-related impairment of normal homeostatic mechanisms result in a persistent activation, which is associated, for example, to a robust induction of oxidative stress (Figures 4, 5; von Bernhardi, 2007; Herrup, 2010), or to the upregulation of NFκB. In fact, NFκB response is age-dependent, and it is another candidate for age-dependent changes due to its role in the regulation of immunity, inflammation, and cell death (Adler et al., 2007). Blockade of NFκB in aged mice has been reported to reverse the gene expression program and cell morphology, “rejuvenating” old mice (Adler et al., 2008). TNF*α* signaling involves NFκB, resulting in a beneficial or detrimental response depending on the age and the type of stimuli. Stimulation of 24 month-old rat neurons with TNF*α* plus Aβ is toxic, whereas those same stimuli are protective for 10 month-old neurons (Patel and Brewer, 2008). The down-regulation of TNFs receptors TNFR1 and TNFR2 signaling observed in aging results in defective NFκB activation and fails to provide a neuroprotective response against Aβ toxicity by TNF*α* (Patel and Brewer, 2008). NFκB accumulates in the nuclei of old neurons; an effect that is also produced by blocking TNFR2. An alternative explanation for the failure of NFκB to activate protective pathways could depend on high concentrations of ROS (Parihar and Brewer, 2007), and the oxidized redox state of aged cells (Parihar et al., 2008). The redox state of NFκB could be a control mechanism regulating its availability (Sulciner et al., 1996). It is unclear whether the over-production of ROS, through a vicious cycle in the aging mitochondria, may activate redox-sensitive NFkB, thereby provoking excessive inflammation in the aged brain (Hayashi et al., 2008; Nakanishi and Wu, 2009; Figure 2).

When exposed to endotoxins like LPS, microglia derived from adult mice secrete high amounts of ROS, whereas young animals microglia predominately produce NO·, with little ROS (Tichauer et al., 2014). Aged microglia become more inflammatory than their younger counterparts upon systemic inflammatory stimulation; thus exacerbating neurodegenerative changes (Combrinck et al., 2002; Cunningham et al., 2005; Godbout et al., 2005; Sierra et al., 2007). Systemic inflammation also causes aged microglia to become more responsive than young microglia, increasing production of inflammatory cytokines (IL1β, IL6 and TNFα). The resulting exacerbated response to inflammatory challenges appears to depend on the priming of microglia by previous activation experience. Primed microglia undergoes a phenotypic shift towards a sensitized state, responding to a secondary “triggering” stimulus more rapidly and robustly than non-primed cells (Harry, 2013). Therefore, the exacerbated response to stimuli of aged microglia can contribute to neuronal damage (Figure 5) and the onset of chronic diseases (Perry et al., 2003, 2007; Perry, 2004).

Age-related changes on cell response involve changes on microglia receptors (Figures 2, 4). Aged microglia show upregulation of Toll-like receptors (TLRs), and TLR4 co-receptor CD14 (Letiembre et al., 2007), as well as age-related changes in signal transduction of TLR4. There are changes in the expression profile of scavenger receptors (SRs; Yamamoto et al., 2002; Hickman et al., 2008). TLRs, CD14, and SRs are pattern recognition receptors (PRRs), key participants of the host defense response and the phagocytosis of pathogen-associated molecules pattern (PAMPs) and damage-associated molecules pattern (DAMPs), being crucial for the innate immune response The activation of these receptors by diverse ligands is associated with activation of microglial cell (Godoy et al., 2012; Murgas et al., 2012, 2014), production of inflammatory mediators, and uptake of pathogens and macromolecules, including Aβ (Alarcón et al., 2005). Thus, changes on their expression pattern affect cell activation (Cornejo and Von Bernhardi, 2013). In addition, aged microglia also express some surface antigens that are not normally expressed by their young counterparts, including the major histocompatibility complex II (MHCII), associated with antigen presentation, and ED1, the rodent equivalent of CD68, associated with phagocytosis. Regardless of the increased CD68, aged microglia are not better phagocytes than young microglia (Floden and Combs, 2011). In fact, aged microglia appear to have a decreased ability to phagocytose Aβ compared with microglia from young mice (Floden and Combs, 2011). We observed that although basal phagocytosis by microglia obtained from 1-year old mice is slightly increased compared with young mice, phagocytosis fails to be induced by TGFβ (Tichauer et al., 2014) or LPS (Cornejo et al., 2014), and is not coupled to an effective clearance machinery (Figure 5). Moreover, in addition to phagocytosis, protein homeostasis is impaired at several levels, including chaperone-mediated protein folding and stability, protein trafficking, protein degradation and autophagy. A major consequence of these impairments is the aggregation of abnormal proteins, which is an important neuropathological finding in several neurodegenerative diseases, such as Parkinson’s disease (PD) and AD (Taylor and Dillin, 2011). Taken together, age-related changes in receptors expression could account for alterations observed in microglial cell function, providing insight on cell phenotypes that could play a role in the pathophysiological changes leading to neurodegenerative diseases.

Autophagy capacity can regulate mitochondrial integrity, ROS production, and subsequent NLR family, pyrin containing 3 (NLRP3) inflammasome activation (Nakahira et al., 2011; Zhou et al., 2011; Salminen et al., 2012). NLRP3 activation is negatively regulated by autophagy, because damaged mitochondria producing high amounts of ROS are removed by autophagy. In fact, inhibition of autophagy triggers accumulation of damaged mitochondria (Zhou et al., 2011), which produce more ROS.

Mitochondrial DNA (mtDNA), which encodes components of the mitochondria electron transfer complexes, is highly susceptible to ROS-mediated damage, due to its close proximity to the ROS generated by the respiratory chain and to its decreased number of protective histones and DNA-binding proteins. Aging-related accumulation of mtDNA damage results in a reduced expression of mitochondria electron transfer complexes, in especial complexes I and IV, because they contain a relatively large number of mtDNA-encoded subunits. The reduced activity of complex I further facilitates the generation of ROS (Lin et al., 2002), establishing a vicious cycle (Kang et al., 2007; Figure 2). Most cells have protective mechanisms, depending on enzymatic breakdown or scavenging of ROS (Figure 1). However, antioxidant systems appear to be less functional in the brain, which can lead to persistent increased levels of ROS and RNS reacting with the various target molecules (Halliwell, 2006).

Functional decline of lysosomes and mitochondria in microglia produces an exacerbated generation of ROS and inflammatory mediators, which could further promote microglia aging (Hayashi et al., 2008). Accumulation of mitochondrial DNA oxidative damage in microglia during aging, increases ROS production. The increased intracellular ROS, in turn, activates the redox-sensitive nuclear factor *kappa* B, inducing neuroinflammation (Nakanishi and Wu, 2009), which in turn also promotes oxidative stress. Mitochondria-derived ROS and cathepsin B, are also involved in the microglial production of interleukin-1β (Figure 2).

During aging, autophagy efficiency declines and becomes dysfunctional, resulting in the accumulation of waste materials within cells (Salminen et al., 2012). On the other hand, induction of phagocytosis on LPS-primed microglia can cause lysosomal damage. The release of cathepsin B (CatB), a lysosomal cysteine protease, into the cytoplasm triggers the activation of the NLRP3, leading to the production and secretion of IL1β (Figure 2) and IL18 (Halle et al., 2008; Hornung et al., 2008). Interestingly, a NLRP3-deficient AD mice model show improvement of their spatial memory deficits, a reduced expression of brain caspase-1 and IL1β, and enhanced Aβ clearance (Heneka et al., 2013). In addition of Aβ, cholesterol crystals is also a major causative factor of age-related diseases such as atherosclerosis, and also shows activation of the inflammasome in a CatB-dependent manner (Duewell et al., 2010; Masters et al., 2010).

## Aged Microglia-Related Neuronal Impairment and Neurodegenerative Diseases

Age-dependent changes gradually have a toll on brain homeostasis and function (Herrup, 2010; von Bernhardi et al., 2010), changing glial cell reactivity (von Bernhardi, 2007). Cytotoxic activation of microglia, increased production of inflammatory cytokines, and ROS combined with impaired ability to regulate increased oxidative stress in the aging brain (Conde and Streit, 2006b; von Bernhardi et al., 2010). Those changes appear to be causative factors for neurodegenerative processes, (Figure 5; Block et al., 2007) and the associated decline in motor and cognitive functions (Forster et al., 1996; Navarro et al., 2002).

Chronic inflammation induces deficits in long-term potentiation (LTP), the major neuronal substrate for learning and memory, in middle-aged but not in young rats (Liu et al., 2012). Similarly, *in vivo* microinjection of fibrillary Aβ in the cortex of aged rhesus monkeys showed neurodegeneration, tau phosphorylation, and microglial cell proliferation, but not in young monkeys, suggesting that Aβ neurotoxicity is a pathological response of the aging brain (Geula et al., 1998). In this context, microglia upregulated production of IL1β, is possibly implicated in age-associated cognitive impairments (Rachal Pugh et al., 2001; Maher et al., 2006). As mentioned above, aged microglia actively participate in the genesis of neuronal damage in neurodegenerative diseases, through production of inflammatory mediators and ROS (Block et al., 2007), but also because of the impairment of their neuroprotective functions (Figure 5). Thus, microglia contribute to the death of dopaminergic neurons in PD, forebrain neurons in AD, and motor neurons in amyotrophic lateral sclerosis (ALS; Boillée et al., 2006; Mount et al., 2007). Similarly, TNF*α* promotes PD progression (McCoy et al., 2006), whereas the absence of TNFR1 protects against AD- and PD-like disease in mice (Sriram et al., 2002; He et al., 2007).

Neurodegenerative diseases often have increased generation of RNS and ROS as an early event (Perry et al., 2002; Shi and Gibson, 2007), which can contribute to neuronal cell injury via various redox reactions (Figure 1). Deficiency in antioxidant enzymes, such as superoxide dismutase (SOD), increases disease associated phenomena (Li et al., 2004a), increasing tau phosphorylation (Melov et al., 2007), and amyloid and tau aggregation (Li et al., 2004a), and accelerates behavioral impairment (Esposito et al., 2006). Thus, oxidative damage in the brain of AD patients and animal models is more abundant than that observed in age-matched control individuals. Conversely, increased expression of antioxidant enzymes attenuates AD phenotype (Dumont et al., 2009).

There are additional mechanisms for reactive species-related impairment, NO· target cysteine residues of proteins to form S-nitrosothiols (SNOs). The interaction with proteins that are targets of S-nitrosylation represents NO· signal transduction (Hess et al., 2005). S-nitrosylation switches the on-off functions of receptors, GTPases, and transcription factors, and can affect mitochondrial function. NO· reversibly inhibits complexes I and IV (Clementi et al., 1998), further increasing release of ROS by mitochondria, further promoting dysfunction of mitochondrial dynamics (Bossy-Wetzel and Lipton, 2003; Barsoum et al., 2006). Moreover, S-nitrosylation modulates GTPase activity of the mitochondrial fission protein dynamin-related protein 1 (Drp1), favoring altered mitochondrial dynamics, synaptic damage, and eventually neuronal death (Cho et al., 2009). Other examples relevant for aging and neurodegeneration are: (i) the S-nitrosylation of protein-disulfide isomerase (PDI, an enzyme relevant for the maturation and transport of unfolded secretory proteins), which abolishes PDI-mediated inhibition of neurodegenerative changes triggered by endoplasmic reticulum (ER) stress, misfolded proteins, or proteasome inhibition (Uehara et al., 2006); and (ii) the S-nitrosylation of ApoE, resulting in changes of its interaction with low-density lipoprotein (LDL) receptors (Abrams et al., 2011).

## Microglia and Alzheimer’s Disease

Neurodegenerative diseases, including AD, involve several converging disease mechanisms, generating a functional interplay between neurons and glial cells (Figure 5). The AD brain is characterized by the presence of senile plaques, constituted by aggregated Aβ, and neurofibrillary tangles (NFTs), formed by hyper-phosphorylated tau, as well by synapse and neuronal loss (Uylings and de Brabander, 2002), and glial cell activation (Kim and de Vellis, 2005; Jellinger, 2006; Heneka and O’banion, 2007; von Bernhardi, 2007; von Bernhardi et al., 2010). Interestingly, Alzheimer, on his original descriptions, already stated that these lesions were markers of an upstream process rather than the disease cause (Davis and Chisholm, 1999). The fact that brain innate immune response could be involved in the genesis of neurodegenerative diseases (Nguyen et al., 2002; Björkqvist et al., 2009; von Bernhardi et al., 2010), lead to re-consider the role of Aβ and propose glia to be a leading factor in the pathology of AD (von Bernhardi, 2007). The hippocampus, one of the regions affected early by neurodegeneration in AD, is one of the most densely populated by microglia together with the *Substantia nigra*. However, most scientists who adhere to the “amyloid cascade hypothesis” of AD, view Aβ as the cause of AD and neuroinflammation just as a consequence of glia activation (Akiyama et al., 2000; Heneka and O’banion, 2007; Hirsch and Hunot, 2009).

Microglia are intimately associated with Aβ plaques in AD, but not with the diffuse Aβ plaques of the normal aged brain (Itagaki et al., 1989; von Bernhardi et al., 2001; von Bernhardi, 2007; Hashioka et al., 2008; Heurtaux et al., 2010). The trigger for microglia activation is unclear, but the invasion of plaques by active microglia has been reported in AD transgenic mice models, when Aβ is injected into the brain or in *in vitro* experiments (von Bernhardi et al., 2001; Alarcón et al., 2005; Reed-Geaghan et al., 2009; Njie et al., 2012; Thanopoulou et al., 2010). Their activation by Aβ (Simard et al., 2006; Hashioka et al., 2008; Koenigsknecht-Talboo et al., 2008) results in cell transformation (Husemann et al., 2001). Microglia aging is associated with several mechanisms underlying the formation and accumulation of Aβ aggregates. Microglia clearance (phagocytosis plus degradation) of Aβ is reduced leading to its initial accumulation (Floden and Combs, 2011; Zhao et al., 2014), as well as its capacity to migrate (Sheng et al., 1998; Damani et al., 2011) and shift among inflammatory activation patterns towards a more phagocytic stage (Sierra et al., 2007; Streit et al., 2009; Schuitemaker et al., 2012). Similar results have been reported on AD patients (Mawuenyega et al., 2010). There is an age-related impairment of phagocytosis (Harry et al., 2000; Zhao et al., 2014) and clearance. Clearance by both microglia and astrocytes appears to depend on peroxisome proliferator-activated receptor-γ (PPARγ) and apolipoprotein E (apoE) levels, which promote the proteolytic clearance of soluble forms of Aβ (Mandrekar-Colucci et al., 2012). In addition, human genetic studies indicate that coding variants of TREM2, a regulator of microglia activation and phagocytosis, are suggestive of microglia immune senescence (Guerreiro et al., 2013), and results in a substantial risk for AD. Plaque-associated reactive microglia in these animals show enhanced staining for TNF*α* and IL-1β (Benzing et al., 1999). Neuroinflammation as well as other stressors promote production and release of Aβ (Lee et al., 2007; Mosher and Wyss-Coray, 2014) as well as its amyloidogenicity, favoring its aggregation. However, acute increased levels of various inflammatory factors, including IL1β and IL6 are associated with activation of glial cells and reduced amyloid pathology (Chakrabarty et al., 2010; Jiang et al., 2015), although chronic neuroinflammation fails promoting amyloid removal. Promotion of Aβ production and aggregation has been also observed secondary to microglia-related ROS through a stress response or depending on oxidative modifications of the peptide (Giasson et al., 2002).

Importantly, Aβ is also clearly indicated as a source of oxidative stress (Varadarajan et al., 2000), as Aβ activates microglia to produce extracellular superoxide radical (O_2_·-; Qin et al., 2002; Bamberger et al., 2003), and can be a potent inducer of NFκB via the induction of intracellular ROS (Lee et al., 2005; Valerio et al., 2006) as well as through the TNFR1 signaling, which results in neuronal apoptosis (Li et al., 2004b; Valerio et al., 2006).

**Figure 6:**
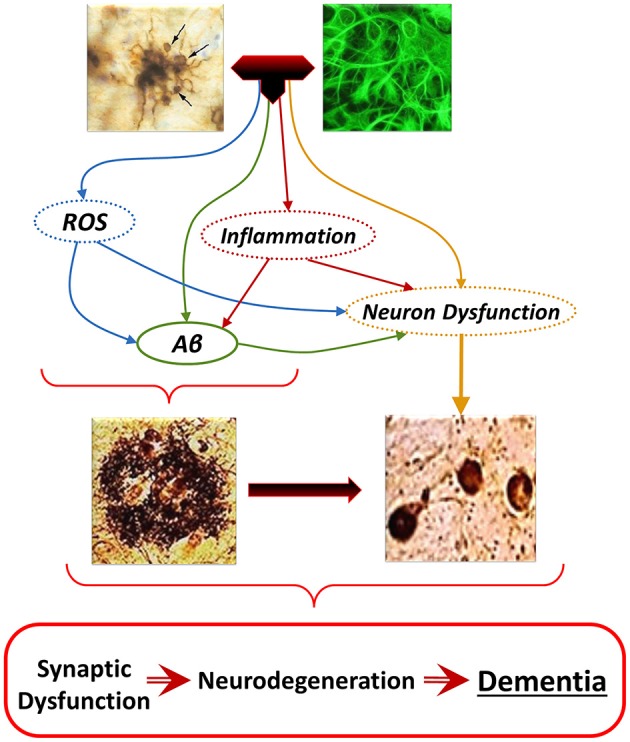
**The “Glial Cell Dysregulation Hypothesis” of Alzheimer’s disease (AD).** The glial cell dysregulation hypothesis proposes that AD has its cause on changes on the activation of microglia and on impaired regulation, which become increasingly cytotoxic decreasing their defensive functions. Impaired activation results in oxidative stress, persistent neuroinflammation and neuronal dysfunction, all of which can also induce production and aggregation of Aβ, and additional neuronal dysfunction. Inflammatory activation, secondary to aging and to various forms of stimuli or injury through life, can result in glial cell dysregulation. Dysregulated activation of glia, through the abnormal release of cytokines, reactive species, and other mediators, contributes to the increased expression of Aβ as well to functional and degenerative changes of neurons, perpetuating abnormal activation of glia, synaptic dysfunction and cell damage.

In addition to the role of oxidative stress in neuron dysfunction and degeneration, secondary to Aβ neurotoxicity, excitotoxicity, aggregation of proteins, and impaired calcium metabolism (Kuchibhotla et al., 2008; Lopez et al., 2008; Santos et al., 2010a,b), ROS appears to be a common mediator unifying the spectrum of cellular mechanisms leading to AD (Figure 6). Oxidative damage of the brain of AD patients and animal models include lipid peroxidation (Praticò et al., 1998; Butterfield and Lauderback, 2002; Butterfield, 2002; Butterfield et al., 2002), and oxidation of proteins and nucleic acids (Nunomura et al., 2001, 2004). RNA and DNA oxidation could impair protein synthesis, DNA repair, and transcription, and could eventually lead to cell death (Figure 1; Ding et al., 2006). Oxidation of mtDNA is 10-fold more abundant than that of nDNA. Increased mtDNA oxidation could lead to the reported mitochondrial abnormalities, which may contribute to the increase of O2·- leakage, ultimately resulting into elevated oxidative stress (Swerdlow, 2007; Swerdlow et al., 2010).

Glia actively promote neuronal dysfunction and neurodegeneration (von Bernhardi, 2007) through oxidative stress mechanisms by: (i) modifying intracellular proteins and lipids (Lovell et al., 2001; Halliwell, 2006; Zhu et al., 2007); (ii) inducing mitochondrial dysfunction, which increases production of ROS, and activates caspases, activating cell death pathway (Baloyannis, 2006; Lin and Beal, 2006a,b) and ATP depletion (Baloyannis, 2006); (iii) facilitating formation of ubiquitinated aggregates of misfolded proteins (Oddo, 2008) as consequence of the impairment of energy-dependent ubiquitin–proteasome pathway and abnormal phosphorylation of cytoskeleton components (Arnaud et al., 2006); (iv) inhibiting glial cell excitatory amino-acid transporter 2 (EAAT2) activity (Tian et al., 2010) inducing release of glutamate by astrocytes (Lauderback et al., 2001). Overactive glutamate receptors increase intracellular free calcium, causing mitochondrial toxicity (Mahad et al., 2008; Kawamata and Manfredi, 2010) and affect several calcium-dependent enzymes leading to dysfunction and initiation of apoptosis (Mattson and Chan, 2003); and (v) activating microglia (Figure 4) and astrocytes to produce and release inflammatory cytokines (von Bernhardi, 2007; Agostinho et al., 2010; Lee et al., 2010; von Bernhardi et al., 2010) and other reactive mediators (NO·, ROS; Zhu et al., 2007; von Bernhardi, 2007; Block, 2008; Agostinho et al., 2010; von Bernhardi et al., 2010). These factors activate signaling pathways of cytokines as well as of eicosanoids produced by cyclooxygenase-2 (COX-2; Wang et al., 2004; Trepanier and Milgram, 2010). Aging and AD also present changes in enzymes involved in glutathione (GSH) metabolism (Figure 1; comprehensive view on glutathione peroxidase (GPx), in Toppo et al. (2009). glutathione S-transferase (GST) activity is decreased in the AD amygdale, hippocampus, parietal lobe, and nucleus basalis of Meynert (Lovell et al., 1998). Decreased glutathione S-transferase omega-1 (GSTO1; Li et al., 2003), can be involved in the activation of IL1β (Laliberte et al., 2003), a fundamental component in the early inflammatory response of AD (Grimaldi et al., 2000; Griffin and Mrak, 2002).

Recapitulating, we consider that neurodegenerative changes in AD are consequence of “mis-activated”, dysfunctional microglia, proposing the “glia dysregulation hypothesis” (Figure 6; von Bernhardi, 2007). The innate immune response, normally protective, becomes abnormally activated, contributing to cytotoxicity (Figure 5; Nguyen et al., 2002; Wyss-Coray and Mucke, 2002; Saud et al., 2005; von Bernhardi, 2007). Normally activated microglia are important as the scavenger cells of the CNS. However, if they fail responding to their normal regulatory feedback and/or they show an impaired ability to clear Aβ (Paresce et al., 1997; von Bernhardi, 2007), glial cells could become predominantly cytotoxic. The distinction is relevant when developing therapeutic approaches. The aim of therapy should be oriented to potentiate a protective pattern of microglial cell function rather than functionally inhibiting microglia as it is most often proposed now.

## Treatment Strategies for Neurodegenerative Diseases

### Modulation of Microglial Cell Activation

Microglia are important actors for maintenance, repair and defense, although dysregulated microglia have deleterious effects. An effective microglia/neuroinflammation based therapy should target regulation of microglial cell response towards a beneficial pattern of activation, rather than their elimination. Because microglial function, as well as the deleterious effect of oxidative damage are associated with the activation of NADPH oxidase and the production of ROS that will act on both intracellular and extracellular targets (Block et al., 2006, 2007), this enzyme complex appears as a relevant therapeutic target. Originally linked only to respiratory burst in phagocytes, over the last decade it has been reported that NADPH oxidase homologues on diverse cells including neurons also play roles in normal function. Several peptides and small molecules, have been reported to inhibit NADPH oxidase, with potential neuroprotective effect over the last decade (Choi et al., 2005; Qin et al., 2005a). Because inhibition of NADPH oxidase activation targets the major generator of high amounts of ROS by microglia, its inhibition would reduce several inflammatory factors, including eicosanoids like PGE_2_ (Wang et al., 2004). The challenge is to develop tools targeting NADPH oxidase isoforms responsible for overproduction of ROS by phagocytes like microglia. The efficacy as neuroprotector of the NADPH oxidase inhibitor diphenyleneiodonium has been reported in both LPS- and 1-methyl-4-phenyl-1,2,3,6-tetrahydropyridine-treated mice (Wang et al., 2015). Diphenyleneiodonium attenuates progressive dopaminergic degeneration, with high efficacy in protecting the remaining neuronal population and restoring motor function even at late stages of disease progression in PD mouse models. Neuroprotection is associated with inhibition of microglial cell activation, decreased α-synuclein aggregation, and reduction of inflammatory mediators (Wang et al., 2015).

Also some inflammatory cytokines have been considered as possible therapeutic targets for AD (Greig et al., 2004; Heneka and O’banion, 2007; Lee et al., 2010). However, a side effect on therapies blocking inflammatory cytokines is the immune suppression caused by these drugs that leaves the patient prone to suffer grave infections. Systemic administration of the anti-inflammatory antibiotic minocycline, which inhibits microglia activation (Kohman et al., 2013a) affects strongly microglia, but also astrocytes, perivascular, meningeal, and infiltrating macrophages. It has been reported that minocycline restores LTP deficits, while normalizing the level of IL1β. These beneficial effects indicate that neuroinflammation could contribute to the deficits in synaptic plasticity, learning and memory observed during normal aging. However, minocycline use reveals the complexity of the effects of microglia function in neurodegenerative disease models. Minocycline show different effects on microglial cell activation and cognitive function along different phases of the life spans of animal models (Kohman et al., 2013a) suggesting that although inhibition of microglia can be beneficial at one stage of disease progression, it becomes detrimental at others.

### Activation of Antioxidant Pathways

Reduction of ROS and oxidative stress could be also achieved through the activation of antioxidant pathways. In addition to the relatively weak antioxidant defenses of the brain, brain aging also determines loss of the endogenous mechanisms of free radical scavenging. Among cellular antioxidant defenses, heat shock proteins have been regarded as cytoprotector for oxidative damage-dependent mechanisms in neurodegenerative diseases. Among the stress proteins, the redox-regulated heme-oxygenase 1 (HO-1) gene, and its activation represents a protective system potentially active against brain oxidative injury. HO-1 polymorphisms have been associated with increased AD susceptibility, and dysregulation of the HO system has been associated with brain aging and the pathogenesis of AD (Markesbery, 1997; Pappolla et al., 1998). AD patients’ brains present microglia recruitment by neurons with tau abnormalities. Those cell clusters correlate with increased levels of NRF2 and HO-1, suggesting an attempt of the diseased brain to limit microgliosis. Microglial cells HO-1 could be especially relevant for the regulation of neurotoxic mediators, being responsible of the antinflammatory effect of compounds such as schizandrin C (Park et al., 2013) and several other compounds (Foresti et al., 2013). Lastres-Becker et al. recently showed that fractalkine activated AKT in microglia, upregulating the transcription factor NRF2, and its target genes including HO-1. Fractalkine regulates microglial cell activation in neurodegenerative diseases. In a mouse model of tauopathy, they confirmed that NRF2- and fractalkine receptor-KO mice did not express HO-1 in microglia and showed they played a crucial role in the attenuation of neuroinflammation. Those observations suggest that NRF2-dependent induction of HO-1 could limit over-activation of microglia (Lastres-Becker et al., 2014). *In vitro* studies report a decreased HO-1 expression in HIV-infected macrophages. HO-1 deficiency correlates with increased glutamate and neurotoxicity, whereas HO-1 siRNA knockdown or its enzymatic inhibition in HIV-infected macrophages increased supernatant glutamate and neurotoxicity. In contrast, induction of HO-1 by dimethyl fumarate (DMF) decreased glutamate and neurotoxicity. Furthermore, increased IFNγ, as observed in CNS HIV infection, reduced HO-1 expression in cultured human astrocytes and macrophages (Gill et al., 2014). There are reports that activation of HO-1 is strongly protective against oxidative damage and cell death in neurons. Thus, modulation of HO-1 should represent a potential pharmaceutical strategy for the treatment of neurodegenerative disorders (Racchi et al., 2008; Schipper and Song, 2015).

### Mitochondrial Antioxidants

Mitochondria have key roles in the production of ROS and in apoptosis signaling. Several compounds targeting mitochondria are currently being tested in clinical trials for treatment of neurodegenerative diseases. Mitochondrial antioxidants appear to be especially interesting at preclinical level (Szeto et al., 2014). Coenzyme Q10 (CoQ10), a carrier of the electron transport chain of oxidative phosphorylation, has been shown to be neuroprotective by attenuating mitochondrial dysfunction and aging (Shetty et al., 2014). However, the fact that these oral antioxidants cross poorly the BBB, has slowed down their therapeutical use; directing new research towards more soluble, shorter chain CoQ10 derivatives, such as idebenone [6-(10-hydroxydecyl-2,3-dimethoxy-5-methyl-1,4-benzoquinone], decylubiquinone (dUb), and MitoQ10. MitoQ10 has the advantage of being accumulated within mitochondria, where it is activated into ubiquinol, which can reduce mitochondrial oxidative damage (Lu et al., 2008). Other class of mitochondrial antioxidants are Szeto-Schiller (SS) peptides (Szeto, 2014), which localize in mitochondria at a broad condition of mitochondrial membrane potential. *In vivo* experiments revealed that SS peptides are protective, increasing survival and motor performance, and decreasing cell death (Moreira et al., 2010). In PD animal models, SS peptides also protect dopaminergic neurons against MPTP neurotoxicity (Moreira et al., 2010).

Therapeutic effects of the regulation of NADPH oxidase and antioxidant treatment will not be restricted exclusively to microglia. However, the development of drugs for specific isoforms and the fact that neuroinflammation is mostly driven by microglia and astrocytes, will have an enormous impact on the cytotoxic activation of glial cells, by reducing both ROS, inflammatory cytokines and endogenous inflammatory mediators.

## Life-Style Changes Prevent Microglia dysrEgulation and Cytotoxic Activation

**Figure 7:**
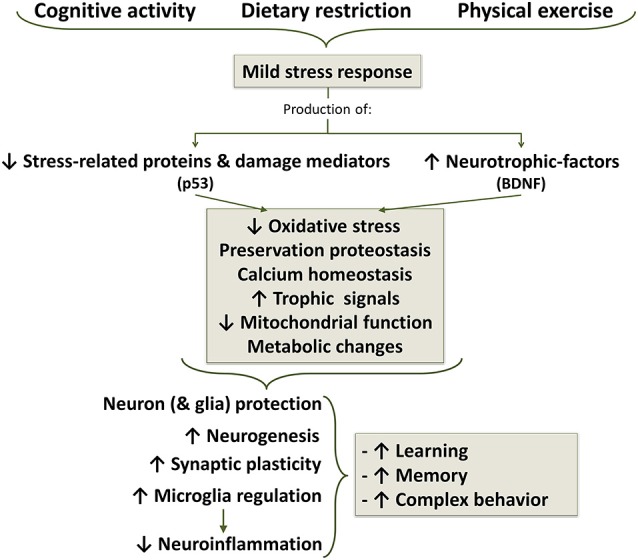
**Life style changes as a strategy for aging well.** Cognitive activity, dietary caloric restriction and moderate physical exercise induce mild stress responses which results in a decreased production of stress proteins and reduction of oxidative stress. In additions, there is an increased production of neurotrophic factors, among which brain-derived neurotrophic factor (BDNF) appears to be one of the most important, but also participate growth hormone (GH) and insulin growth factor 1 (IGF1). Decreased stress signal and increased trophic signal acts on mitochondrial function, improving energetic metabolism and reducing oxidative stress to a protective level. Stress signals and ROS, below a certain threshold concentration, induce survival signals capable of restoring cellular homeostasis but, at higher or continued levels, can contribute to aging and degenerative changes.

Accumulating evidence show that exercise, dietary restriction, cognitive intervention (enriched environment) as well as other mild stressors can play a role in reducing microglial activation and priming during aging (Figure 7). Moderate exercising is capable of even reducing the exaggerated neuroinflammation in response to infection-type of stimuli in aged animals, with its increased cytokine production and cognitive deficit (Barrientos et al., 2011), and age-related microglial sensitization (Barrientos et al., 2011; Kohman et al., 2013b), suggesting that exercise could be an effective intervention to prevent microglial cell aging. Furthermore, In adult APP/PS1 mice, exercise increase neurobehavioral performance, which is associated with increased numbers of certain populations of cholinergic and serotoninergic neurons, and reduced Aβ levels and microglia activation (Ke et al., 2011). Beneficial effects of exercise and cognitive intervention could, at least in part, result from its induction of brain-derived neurotrophic factor (BDFN; Barrientos et al., 2011; Polito et al., 2014). Although most of reports are related to the effect of BDNF on neuron function and survival, there are reports on its effect on inhibiting activation of microglia (Garofalo et al., 2015). Dietary restriction also appears to attenuate age-related activation of microglia, resulting in beneficial effects on neurodegeneration and cognitive decline (Morgan et al., 2007). It has anti-inflammatory and anti-apoptotic effects (Loncarevic-Vasiljkovic et al., 2012), and has been shown to elicit many health promoting benefits, delaying immunosenescence and attenuating neurodegeneration in animal models of AD and PD. However, the mechanisms involved in the effect of dietary restriction on microglial cell activation are poorly understood. Exposure to dietary restriction attenuates LPS-induced fever, and LPS-induced microglial activation in some specific brain regions, including the arcuate and ventromedial nuclei of the hypothalamus and the subfornical organ. Activation of microglia in the hypothalamic nuclei was positively correlated with body temperature (Radler et al., 2014). Dietary restriction suppresses LPS-induced secretion of inflammatory cytokines, and shifts hypothalamic signaling pathways to an anti-inflammatory bias (Radler et al., 2015).

Interestingly, both exercise and dietary restriction have been recently shown to promote mitochondrial biogenesis and expression of mitochondrial transcription factor A (TFAM) in the rat brain (Picca et al., 2012; Zhang et al., 2012). Collectively, exercise, cognitive activity, and dietary restriction could be effective ways to slowdown brain aging by preventing microglia aging through secretion of growth factor and regulatory cytokines. Although those effects are not restricted to microglia, the fact that microglia are the major drivers of neuroinflammation, determines that interventions affecting them can have an enormous impact on the brain homeostatic response.

## Concluding Remarks

Aging is a major risk factor for the great majority of neurodegenerative diseases. Age dependent changes, including increased glial cell activation, neuroinflammation, oxidative stress, impaired mitochondrial function, and impaired protein processing, could lead to the dysregulation of microglial cell functions resulting, among several alterations in cytotoxicity and accumulation of Aβ, generating the hallmark histopathology of AD. Whereas each of these age-dependent changes are discreet in the normal aging process, their combined effect, together with the genetic background and environmental conditions could initiate the vicious circle of cytotoxic activation (von Bernhardi, 2007). Participation of oxidative stress could be both a trigger and a consequence of Aβ accumulation, mitochondrial impairment, cytotoxic activation of microglia, proteasome dysfunction and protein misfolding, contributing to the potentiation of the other disease mechanisms. Additionally, oxidative stress, cytotoxicity and Aβ aggregation further decrease proteasome activity, creating a vicious circle leading to more Aβ and tau aggregation.

Microglia, in a close crosstalk with astrocytes, neurons and other brain cells, serve crucial functions as the scavenger system of the CNS, providing beneficial functions as tissue repair in the CNS. However, chronic, dysregulated activation of microglia appears to lead to deleterious effects inducing malfunction and damage of brain cells. What drives this dysregulation is not fully understood, but age-related impairment of regulatory mechanisms, as observed for TGBβ transduction signaling (Tichauer et al., 2014) are a promising hypothesis for understanding cytotoxic activation in aged individuals (von Bernhardi et al., 2011). Nonetheless, despite the undeniable potential of activated microglia to become deleterious, microglia have a profound immune-modulatory and reparative potential in the CNS. Thus, instead of abolishing microglia activation as it is most often proposed, strategies to potentiate those beneficial functions while inhibiting cytotoxic activation should be developed. Such strategy may well constitute the way to treat neurodegenerative disorders, but demands a better understanding of the protective and modulatory pathways of immune activation. Additional research is needed for the identification of new pathways that may decrease the impact of microglial cell dysfunction, in order of breaking the vicious circle leading to neurotoxicity.

Further research is necessary to develop effective pharmacological interventions against brain aging. Most of the proposed targets, antioxidants, anti-inflammatory drugs affecting cytokines, and microglia inhibitors, deeply affect physiological cell signaling and functions, including pro-survival signaling pathways, resulting in unacceptable side effects. In that perspective, multi-target pharmacological approaches aimed to reestablish normal regulation of microglia in the aged brain may be future research avenue for slowing senescence-related impairment. Furthermore, non-pharmacological strategies, like exercise, life style changes and dietary restriction, could promote a healthy aging through their effects on promoting microglial physiological functions, while reducing inflammation and ROS production.

## Conflict of Interest Statement

The authors declare that the research was conducted in the absence of any commercial or financial relationships that could be construed as a potential conflict of interest.
